# The importance of providing counselling and financial support to patients receiving treatment for multi-drug resistant TB: mixed method qualitative and pilot intervention studies

**DOI:** 10.1186/1471-2458-14-46

**Published:** 2014-01-17

**Authors:** Sushil C Baral, Yeshoda Aryal, Rekha Bhattrai, Rebecca King, James N Newell

**Affiliations:** 1 Health Research and Social Development Forum, PO Box 24133, Thapathali, Kathmandu, Nepal; 2 Nuffield Centre for International Health & Development, Leeds Institute of Health Sciences, University of Leeds, 101 Clarendon Road, Leeds LS2 9LJ, UK

## Abstract

**Background:**

People with multi-drug resistant tuberculosis (MDR-TB) in low-income countries face many problems during treatment, and cure rates are low. The purpose of the study was (a) to identify and document the problems experienced by people receiving care for MDR-TB, and how they cope when support is not provided, to inform development of strategies; (b) to estimate the effectiveness of two resultant strategies, counselling alone, and joint counselling and financial support, of increasing DOTS-plus treatment success under routine programme conditions.

**Methods:**

A mixed-method study comprising a formative qualitative study, pilot intervention study and explanatory qualitative study to better understand barriers to completion of treatment for MDR-TB. Participants were all people starting MDR-TB treatment in seven DOTS-plus centres in the Kathmandu Valley, Nepal during January to December 2008. The primary outcome measure was cure, as internationally defined.

**Results:**

MDR-TB treatment caused extreme social, financial and employment hardship. Most patients had to move house and leave their job, and reported major stigmatisation. They were concerned about the long-term effects of their disease, and feared infecting others. In the resultant pilot intervention study, the two strategies appeared to improve treatment outcomes: cure rates for those receiving counselling, combined support and no support were 85%, 76% and 67% respectively. Compared with no support, the (adjusted) risk ratios of cure for those receiving counselling and receiving combined support were 1.2 (95% CI 1.0 to 1.6) and 1.2 (95% CI 0.9 to 1.6) respectively. The explanatory study demonstrated that patients valued both forms of support.

**Conclusions:**

MDR-TB patients are extremely vulnerable to stigma and extreme financial hardship. Provision of counselling and financial support may not only reduce their vulnerability, but also increase cure rates. National Tuberculosis Programmes should consider incorporating financial support and counselling into MDR-TB care: costs are low, and benefits high, especially since costs to society of incomplete treatment and potential for incurable TB are extremely high.

## Background

Globally, the number of people developing multi-drug resistant TB (MDR-TB) is increasing, with an estimated 650,000 cases in 2010 [1]. In response, WHO and its partners developed the DOTS + strategy [2], which requires MDR-TB patients to take multiple powerful antibiotics with daily direct observation of treatment (DOT) for a minimum of 20 months. MDR-TB treatment at sites using international standards of care was successful only in 69% of cases [3]. A systematic review of MDR-TB treatment outcomes found an overall default rate of 12% (95% CI 0.1% to 36%) using standardized regimens [4]. Patients who start but do not complete MDR-TB treatment are likely to die or may develop extensively drug resistant TB (XDR-TB), which is considered to be virtually untreatable and a serious public health threat worldwide [5-7].

In low- and middle-income countries, daily DOT places a major burden on patients. Furthermore, patients need regular access to the scarce technical expertise required to provide regular check-ups including for potentially life-threatening drug side-effects. Thus patients must move near a centralized DOTS-plus centre for at least 20 months, meaning they are unable to continue their employment, have to pay substantially more for accommodation and food, and lose their normal family support networks.

In our work with people with MDR-TB in Nepal [8], it became clear that additional support was necessary [9]. The objectives of our study were therefore (a) to identify and document the problems experienced by people receiving care for MDR-TB, to inform development of strategies, and (b) to estimate the effectiveness of two resultant strategies.

The setting for our study was Nepal, a mid-TB burden country, with a well functioning National TB Programme (NTP). In Nepal, terrain causes problems of health service provision [10] and TB is highly stigmatised [11]. Nepal has been a DOTS + pilot country since November 2005, with non-completion rates of 22%, 15% and 18% in 2005, 2006 and 2007 respectively [12].

## Methods

We performed a mixed-method study including formative and explanatory qualitative components and a pilot intervention study at the seven DOTS-plus centres that existed in the Kathmandu Valley at the time of the study. Prior to the start of the formative study, we randomly allocated the DOTS-plus centres to 3 types of care – 2 to counselling, 3 to combined support, and 2 to usual care – by selecting randomly from the numbers 1 to 7. Individual randomization of patients was infeasible because of the certainty of contamination and consequent disquiet among patients.

### Formative study

Using purposive sampling from MDR-TB registers at the 5 intervention centres, we identified 49 registered people with MDR-TB for interview, the sample size being based on time and resources available: all agreed to take part. Interviews took place in the patient’s DOTS-plus centre. The interviews were conducted in Nepali by SCB, YA and a public health nurse with training in patient counselling: all three had experience in in-depth interviewing. Interview guides were developed by SCB and YA based on their experience with people with MDR-TB. Areas explored included: socio-demographic characteristics; employment history; sources of income; living costs; reason(s) for relocation (non-local residents only) and relocation costs; knowledge of MDR-TB including communicability and curability; knowledge and experience of drug side effects and how they were addressed; care-seeking practices during diagnosis and treatment; distance to MDR-TB centre; travel costs; interactions with family, friends and others; and implications of daily visits to the DOTS-plus centre. Interviews were recorded and notes taken. Interviews lasted between 45 and 60 minutes.

The data were coded by SCB and YA, and analysed thematically using a framework approach, combining a-priori and emergent themes. At the start of the coding process, SCB and YA read the initial transcripts and generated preliminary codes. Repeated discussion, reasoning and reflection during the coding process led to a well-defined set of codes. The two researchers then coded the remaining transcripts. When necessary, new codes were discussed and agreed upon and the code set was updated accordingly.

### Pilot intervention study

We compared three groups – counselling; combined counselling and financial support; and for comparison, usual care (no support). All MDR-TB patients starting treatment at the DOTS-plus centres from January-December 2008 were eligible for inclusion.

Patients receiving counselling were counselled individually and in small groups by a Public Health Nurse who was trained to provide counselling for this research. Counselling was provided every 2–3 weeks across all sites. The counsellor and researchers met regularly to discuss issues raised during counselling.

Patients receiving financial support were given Nepali Rupees (NRs) 2000 (US$ 28) per month: this was meant to cover local transport, food and rental costs, but patients were free to use it as they chose.

Based on an assumed usual care cure rate of 70%, and not allowing for potential effects of clustering, we estimated that 62 patients were required in each group to attain 80% power, testing at the 0.05 level (2-sided), to detect a difference in cure rate of at least 20% between either of the interventions and usual care. We made no allowance for loss to follow-up, since loss to follow-up was categorised as default. We obtained data on age and sex for each patient from routine health service records. In the two intervention groups, we collected additional socio-economic data at enrolment. Patients not receiving support were not asked for additional data, as to do so might have started to approximate or resemble counselling, and thus reduced our ability to observe additional effects of counselling. The epi.2by2 function of the R [13] “epiR” package [14] was used to estimate unadjusted and adjusted risk ratios.

### Explanatory study

In the two groups that included counselling, we collected additional qualitative information on problems that patients experienced. We used a similar approach to data collection and the same sample (27 receiving combined support and 22 counselling support) as in the formative study. Areas explored included: experience with family members, relatives, friends, community members, and at the workplace before and after MDR-TB; impact of illness on general living and employment; mechanisms for coping with MDR-TB and other associated illness; experience with the health system especially in routine management of illness; management of drug side effects; support from the health system, service providers, family, relatives, at the workplace and in the community; disclosure of their disease status to others; problems/issues experienced by the respondents and their management; and perceptions of the interventions. Data was analysed using the procedures described above for the formative study.

Ethics approval was granted by the Nepal Health Research Council.

## Results

All eligible patients agreed to take part in each of the three parts of the study, and all participants gave verbal informed consent.

### Results from the formative study

The formative study aimed to explore patients’ explanations of the impact of MDR-TB and its treatment. These impacts can be categorised into three broad categories.

#### Social and psychological impacts

Respondents experienced substantial enacted stigma, leading to divorce, cancellation of impending marriages, breakdown of family relationships, and isolation within the family. Most reported that neighbours harassed them both directly and through unpleasant gossip and many experienced hatred or avoidance by friends and neighbours. Landlords evicted patients from, or rejected applications for, rented accommodation. Many experienced discrimination in public places. Health workers were often accused of discrimination.

“Hotels and cafes won’t let me use their plates: I have to take my own.”

“I was evicted from my rented room. The house owner blamed me for the death of her son: she thought I transmitted my disease to him.”

This discrimination was seen partly because of fear of being infected, although respondents thought TB was sometimes used as a concrete excuse to discriminate against more intangible attributes such as dislike. However, many respondents reported that once their sputum tests became negative, and they looked healthy again, they got better responses from family, friends and neighbours.

Many respondents also reported perceived stigma. Some hid their disease and isolated themselves for complex reasons combining fear of discrimination and fear of infecting others. A few believed it was good to relocate for treatment to a place where they were not known, because of the reduced risk of disease disclosure, but found managing costs of relocation very difficult.

Respondents felt guilt leaving their families while they received treatment, not only because they were unable to maintain their contribution, but because they became major burdens. Some regarded MDR-TB as divine punishment for misdeeds, exacerbating feelings of guilt.

“I feel guilty because I got married a few months before getting diagnosed with MDR-TB. I ruined her life.”

Because they had to relocate to receive treatment, many respondents lost the support of their family and community. Isolation led to misery, boredom and introspection. Some felt suicidal because of the burden they placed on the family.

“There’s no-one here to help. I wish I could be with my family during this difficult time.”

“If the tests come back positive this time, I’ll kill myself – there’s no point being alive like this.”

Most respondents recommended expanding services so that patients could take treatment at home. They believed that this would speed recovery. Some patients expressed a desire or compulsion to return to their homes even though this meant missing treatment.

“Once I visited home and felt very happy and relaxed to be with my grandchildren. I feel that it would be good to be able to take [DOTS-plus] treatment at home.”

#### Employment, educational and financial impacts

Most respondents had to leave their job and did not find re-employment, causing major hardship. This occurred primarily because they had to relocate to access the DOTS-plus Centre, and found it difficult to get re-employment because they had to visit the DOTS-plus Centre daily. The very few who continued working reported that their illness lowered their performance, leading to employers’ dissatisfaction and consequent feelings of humiliation. Students were not able to perform as before due to impaired intellect and memory, leading to feelings of guilt. Younger people were very worried, as they perceived their counterparts’ careers progressed while theirs stagnated. Relocation costs were high, including rent and daily travel to the DOTS-plus Centre; and substantially higher food and other costs than usual.

“I’m in a big financial mess: I had to leave my job and my debts are increasing day by day.”

Often, patients coped by getting financial support from their family, borrowing money from friends or relatives, or taking a loan. They also sold long-term means of survival such as land, animals and equipment (one respondent sold his vegetable-selling cart) and other assets such as jewellery dowries. Often children were taken out of school and sent to work, to save school fees and generate income.

“I took a loan to come to the city. I don’t know when I“ll be able to repay it as I have no income.”

“I only eat twice a day, have sold my animals and land, and taken out a loan.”

#### Health impacts

Patients had major worries relating to their health. They were unclear about the curability of their disease, and feared relapse after treatment completion. They worried about the effects of MDR-TB drugs and the disease itself. They were concerned about transmitting the disease: many had experience of several people with TB in one family.

“I doubt the curability of my disease. I was told my TB would be cured if I took the medicine regularly, but I took it regularly, and even now I’m taking it regularly, but I’m still not cured [and my TB has returned]. I think I’ll die due to the disease” (relapsed MDR-TB patient).

“I feel like a victim.”

Their frustration was increased because they had multiple problems.

“I am upset having to take so much medicine, not having a job, feeling bored because of having no work, and being a long time away from home, as well as spending without any income and with no other earner in the family.”

A substantial majority of respondents encountered side-effects of MDR-TB drugs. Most consulted the DOTS-plus Centre, but the side-effects continued to disrupt daily DOT and hinder their daily activities. Two reported mental effects so severe that they were arrested and jailed. Some wanted to stop treatment because of the side effects.

“I was jailed, and still have scars from the beating the police gave me. Initially I had great difficulty in taking the medicine.”

“It’s the medicine rather than the disease itself that makes life so difficult.”

Our conclusion from this formative study was that MDR-TB patients would probably benefit from counselling and/or financial support, and thus developed a pilot intervention study with three arms – counselling; combined counselling and financial support; and for comparison, usual care (no support).

### Results from the pilot intervention study

Combined counselling and financial support was provided by 3 DOTS-plus centres: a government clinic (National TB Centre) which acts as a national referral centre for MDR-TB; a semi-governmental hospital (Patan Hospital) with dedicated personnel for lab and clinic management; and a non-governmental organisation (Helping Hands) providing basic MDR-TB treatment services. Counselling was provided by two clinics: a community based hospital (Stupa Hospital) that mainly caters for migrants; and a government clinic located within a central level hospital (Bir Hospital) with a wide range of health services. Clinics providing usual care (no support) were: a teaching hospital (Nepal Medical College) with an in-patient facility and other specialised care; and an NGO run clinic (NATA/GENETUP) with advanced TB diagnosis and treatment facilities.

Lower than expected enrolment in our study DOTS-plus centres due to (limited) expansion of centres in Nepal meant we enrolled only 33 patients to receive counselling and 42 to receive combined support. As partial compensation, we increased numbers on the no-support group to 81, to reduce the expected width of confidence intervals of comparisons. All eligible individuals consented to participate in the study.

Distributions of sex and age were similar across all three groups (Table 1); and in addition distributions of marital status, occupation, origin and residence (for migrants only) were similar across the two intervention groups (Table 2). Most were from the economically productive age group, but were not working at the time of interview. Most had moved for treatment and were staying in a rented room. Farming was the most common source of income. Median incomes and outgoings of the counselling and combined support groups were similar: annual family cash income was NRs 72,000 (US$ 1,028); annual rent paid by participants staying in rented accommodation was NRs 18,000 (US$ 257); annual food costs were NR 68,000 (US$ 971); and average cost of travel for DOT was NRs 7,000 (US$ 100).

**Table 1:** General characteristics of study participants

	**Type of support provided**					
	**Counselling**		**Combined**		**None**	
	**N**	**(%)**	**N**	**(%)**	**N**	**(%)**
**Sex**						
Male	20	(61)	30	(71)	51	(63)
Female	13	(39)	12	(29)	30	(37)
*Total*	*33*	*(100)*	*42*	*(100)*	*81*	*(100)*
**Age group**						
≤20	3	(9)	6	(14)	12	(15)
21-30	19	(58)	13	(31)	33	(41)
31-40	3	(9)	7	(17)	16	(20)
41-50	6	(18)	8	(19)	14	(17)
51-60	2	(6)	5	(12)	3	(4)
>60	0	(0)	3	(7)	3	(4)
*Total*	*33*	*(100)*	*42*	*(100)*	*81*	*(100)*

**Table 2:** Additional characteristics of patients receiving support

	**Type of support provided**			
	**Counselling**		**Combined**	
	**N**	**(%)**	**N**	**(%)**
**Marital Status**				
Unmarried	19	(58)	18	(43)
Married	14	(42)	23	(55)
Other (d/w/s)	0	(0)	1	(2)
*Total*	*33*	*(100)*	*42*	*(100)*
**Occupation**				
Unemployed	18	(55)	38	(91)
Labourer	2	(6)	1	(2)
Housewife	3	(9)	0	(0)
Business	2	(6)	0	(0)
Student	4	(12)	3	(7)
Agriculture	2	(6)	0	(0)
Service	1	(3)	0	(0)
Other	1	(3)	0	
*Total*	*33*	*(100)*	*42*	*(100)*
**Origin**				
Local	10	(30)	7	(17)
Migrant	23	(70)	35	(83)
*Total*	*33*	*(100)*	*(100)*	*42*
**Residence (among migrants only)**				
Rented room	17	(74)	25	(71)
Friend’s home	1	(4)	2	(6)
Relative’s home	4	(18)	7	(20)
Other	1	(4)	1	(3)
*Total*	*23*	*(100)*	*35*	*(100)*

Table 3 gives treatment outcomes by support group. Cure rates for those receiving counselling, combined support and no support were 85%, 76% and 67% respectively. Compared with no support, the unadjusted risk ratios of cure for those receiving counselling and combined support were 1.3 (95% CI 1.0-1.6) and 1.2 (95% CI 0.9-1.5) respectively. Risk ratios were virtually unchanged when adjusted for sex or age group using the Mantel-Haenszel method.

**Table 3:** Treatment outcomes

**Outcome**	**Type of support**					
	**Counselling**		**Combined**		**None**	
	**N**	**(%)**	**N**	**(%)**	**N**	**(%)**
Cured	28	(85)	32	(76)	54	(67)
Defaulted	2	(6)	6	(14)	15	(19)
Died	1	(3)	2	(5)	8	(10)
Failed	2	(6)	2	(5)	4	(5)
*Total*	*33*	*(100)*	*42*	*(100)*	*81*	*(100)*
Unadjusted and adjusted* risk ratios (RR)						
		**Type of support**				
		Counselling		Combined		
		RR (95% CI)		RR (95% CI)		
*(a) for cure versus other outcomes, taking the No Support group as baseline*						
RR		1.3(1.0, 1.6)		1.2 (0.9, 1.5)		
RR adjusted for sex		1.3 (1.0, 1.6)		1.2 (0.9, 1.5)		
RR adjusted for age group		1.2 (1.0, 1.6)		1.2 (0.9, 1.6)		
*(b) for cure versus default, taking the No Support group as baseline*						
RR		1.4 (1.1, 1.7)		1.1 (0.8, 1.6)		
RR adjusted for sex		1.3 (1.1, 1.7)		1.1 (0.8, 1.5)		
RR adjusted for age group		1.2 (1.0, 1.6)		1.2 (0.9, 1.6)		

*Adjusted using the Mantel-Haenszel method.

Since it was possible that support influenced default but not death or failure, we reanalysed the data excluding patients whose outcome was death or failure. Cure rates were 93%, 84% and 78% respectively. Unadjusted risk ratios of cure for those receiving counselling and combined support were 3.9 (95% CI 0.8-18.2) and 1.4 (95% CI 1.1-1.7) and 1.1 (95% CI 0.8-1.6) respectively. Again, risk ratios were largely unchanged when adjusted for age group using the Mantel-Haenszel method.

### Results from the explanatory study

#### Social and psychological impacts

Those receiving counselling took some time to become comfortable with the counsellor and talk openly. Many wept and became very emotional while sharing their problems. They reported that they liked having someone to listen to their problems and give support without discrimination. Their fear was reduced by discussing information about the disease and its treatment. Negative thoughts reduced and self-esteem increased. The counsellors were described as being like good friends in difficult times, especially by those staying alone in rented accommodation.

“… a big relief to talk to you …”

“… it would be good if it [counselling] continues as otherwise there is no one who listens to us.”

Those receiving combined support found it very helpful and believed all patients should receive it. Initially some patients did not like visiting the DOTS-plus Centre but this attitude changed as they shared problems with counsellors. They reported that the support increased their self-esteem and their belief that they would be cured, and decreased their worries. Many patients stated that the support had given them a new life. Those receiving combined support seemed to appreciate counselling more than those only receiving counselling: this seemed to be because counselling worked better when they were not overwhelmed by financial problems.

“… God sent you to provide this support.”

#### Employment, educational, and financial impacts

Although those receiving counselling said their stress was relieved by sharing their problems, they continued to have financial problems that counselling could not fully address. Even through they explicitly said they were happy with the support provided, many missed or were late for counselling because of financial and other problems.

Most of those receiving combined support used the money for food (including food additional to normal consumption), rent and travel costs. Some said they were entirely dependent on the support: many believed they would otherwise have died as there was nothing available for them to eat. Some remitted money to solve financial problems at home. However, some were not able to use the support money as their family took it. Some commented that although the amount given was helpful, more would have been more useful. Unsurprisingly, the financial component was most appreciated by patients who were very poor and had also had to relocate.

“I would have died if I did not get support from you.”

“I get financial support for additional food – but how can I eat it when my children are hungry?”

“I used to eat only once a day. Now I can buy food and pay rent.”

However, a few richer patients did not value the support, seeing it as pocket money. Consequently, such patients did not come on time and did not take counselling seriously.

#### Health impacts

Participants from both groups reported improved understanding of the likelihood of cure, and solved many problems related to their disease and its treatment. These included duration of treatment, management of drugs and understanding of side-effects.

## Discussion

We found that our respondents reported that DOTS-plus treatment caused serious financial and social problems. Financial problems were caused by greatly increased expenses due to having to move near to the DOTS-plus Centre. Social problems included a lack of social support following patients’ removal from their normal social network, stigma, loneliness, and toxic effects of drugs. Since our study started there has been some limited expansion in DOTS-plus provision, including some hostel spaces, which reduces accommodation costs, and minimum treatment duration is now 20 months, but the great majority of patients still need to relocate for nearly two years.

We found that both counselling alone and combined counselling and financial support were valued by patients. In general, we found that those getting combined support were more appreciative of counselling than those receiving counselling alone.

Both forms of support appeared to have a beneficial effect on treatment outcomes, although differences were not significant. This finding is not unexpected, given the problems MDR-TB patients face.

We were unable to find any published studies giving evidence either on the problems people with MDR-TB face during treatment, nor on the effectiveness of interventions to address these problems.

The study has several limitations. The sample sizes of the pilot intervention were less than planned, and did not take clustering (by DOTS-plus centre) into account, so we may not have had the power to detect true effects. Although the three groups were comparable in sex and age distribution, and the two intervention groups were comparable on the other factors recorded, the groups may or may not have been comparable on unrecorded factors: it is difficult to assess the effects of differences in the DOTS-plus centres involved. Analysis did not take clustering into account, due to the limited numbers of clusters. Caution should be taken when generalising the results: in general it can be difficult to generalise qualitative results; and the study was restricted to the Kathmandu Valley.

The study has a number of strengths. The formative studies allowed the key people involved – those receiving treatment for MDR-TB – to express the problems they face in continuing treatment, untainted by the researchers’ preconceptions, and guided development of the intervention. The mixed method approach allowed a clearer interpretation of the formative and pilot intervention studies. Findings accord with anecdotal experiences across many countries.

## Conclusions

We have demonstrated that DOTS-plus treatment causes serious financial and social problems, that both counselling alone and combined counselling and financial support were valued by patients, and that financial and counselling support appear to improve MDR-TB treatment outcomes. Larger studies, and preferably randomised controlled trials (RCTs), are required to confirm our preliminary findings on the effectiveness of these strategies and estimate their cost-effectiveness. However, even before RCTs (which will take at least 4 years) are completed, NTPs should consider how they can support this disadvantaged and vulnerable group of people. Based on this study, the Nepal NTP now gives each MDR-TB patient NRs 1500 (US$ 21) per month during treatment. NTPs should consider doing their own operational research to determine the type(s) of support most appropriate and effective in their context.

## Competing of interests

The authors have no conflicts of interest related to this study or its publication.

## Authors’ contributions

SB was involved in study design, the collection, analysis and interpretation of data, and in the writing of this paper. YA and RB were involved in the collection, analysis and interpretation of data, and in the writing of this paper. RK was involved in the interpretation of data, and in the writing of this paper. JN conceived the study, and was involved in study design, analysis and interpretation of data, and in the writing of this paper. All authors have approved the final version of this paper.

## Pre-publication history

The pre-publication history for this paper can be accessed here:

http://www.biomedcentral.com/1471-2458/14/46/prepub
